# The Conservation Crisis of *Ophiocordyceps sinensis*: Strategies, Challenges, and Sustainable Future of Artificial Cultivation

**DOI:** 10.3390/jof11120892

**Published:** 2025-12-18

**Authors:** Zhoujian He, Meng Ye, Huaxue Wu, Dan Liang, Jie Huan, Yuan Yao, Xinyue Wu, Xiaomei Luo

**Affiliations:** 1 College of Forestry, Sichuan Agricultural University, 211 Huimin Road, Wenjiang District, Chengdu 611130, China; hezhouj@163.com (Z.H.); 17360164303@163.com (Y.Y.); 2 College of Life Sciences, Sichuan University, 29 Wangjiang Road, Wuhou District, Chengdu 610064, China; 15079803161@163.com; 3 Yanyuan County Forestry and Grassland Bureau, Liangshan Yi Autonomous Prefecture, Yanyuan 615715, China; 15680813563@163.com; 4 Baoxing County Natural Resources and Planning Bureau of Yaan City, 256 Lingxiu Road, Baoxing County, Ya’an 625700, China; 2020206034@stu.sicau.edu.cn; 5 Enyang District Agriculture and Rural Bureau of Bazhong City, No. 6, 40 Planning Road, Enyang District, Bazhong 636600, China; 15388135911@163.com

**Keywords:** *Cordyceps sinensis*, resource conservation, in vitro fermentation, market trade

## Abstract

*Ophiocordyceps sinensis*, a fungus revered in traditional Asian medicine, is critically endangered due to climate change and overharvesting. Artificial cultivation is thus essential to meet demand and promote conservation. This review systematically analyses the decline of wild *O. sinensis* and evaluates the two primary cultivation strategies: in vitro mycelial fermentation and in vivo inoculation. We find that in vitro fermentation, while scalable and standardized, yields a chemical profile distinct from that of wild fungi. In vivo inoculation can produce fruiting bodies morphologically and chemically closer to wild specimens but is hampered by technical challenges in host rearing and low infection rates. By dissecting these bottlenecks, this review provides a framework for the sustainable cultivation of *O. sinensis*, crucial for preserving both a cornerstone of traditional medicine and the ecological balance of its native habitats.

## 1. Introduction

*Ophiocordyceps sinensis* (Berk.) G.H. Sung, J.M. Sung, Hywel-Jones & Spatafora, also known as *Cordyceps sinensis*, is commonly referred to as Chinese cordyceps [1]. It is often described as “insect within the grass, and the grass within the insect”, a reflection of its complex biology characterized by the “winter worm, summer grass” phenomenon [2,3]. This intricate biology enables it to produce numerous bioactive compounds beneficial to human health, leading to its widespread use in medicine and as a health supplement [1,4,5]. Due to active constituents such as cordycepin and ISP-1 (a sphingosine analogue), it is frequently employed as an adjuvant immunosuppressive agent for skin and organ transplants [6,7]. Furthermore, bioactive components like cordycepin and polysaccharides have shown potential in cancer treatment [8,9]. β-sitosterol and arachidonic acid have been associated with therapeutic effects against diabetes [2]. Beyond its inclusion in the pharmacopoeias of several Asian countries [10], it is also recognized by the European Union as a food and dietary supplement ingredient, often used by athletes to enhance performance [11]. Notably, Chinese long-distance runners set world records after consuming *O. sinensis* during training [8]. The broad spectrum of applications has consequently led to a significant increase in the demand for *O. sinensis*.

The term “Chongcao (*Cordyceps*)” originates from its morphology as an “insect-fungus complex”, while “Dongchong Xiacao (*O. sinensis*)” is derived from its habit and form, meaning “worm in winter, grass in summer” [12]. Inconsistent understanding of these terms among scholars from different fields has led to some public confusion regarding *O. sinensis* [13]. The *Cordyceps* industry originated from *O. sinensis* and now encompasses multiple species, including *O. sinensis* itself [13]. Wild *O*. *sinensis* is predominantly distributed across four countries surrounding the Tibetan Plateau, where it grows at elevations ranging from 3000 to 5000 m above sea level [5,14]. Specifically, *O. sinensis* refers to the dried complex consisting of the stromata and sclerotia formed by the fungus *Hirsutella sinensis* X. J. Liu, Y. L. Guo, Y. X. Yu & W. Zeng parasitizing larvae of the genera *Hepialus* Viette and *Thitarodes* Fabricius. The annual yield of wild *O. sinensis* is less than 200 tons [14], with market prices for high-quality fruiting bodies reaching as high as US $10,000 to $60,000 per kilogram [15], even exceeding the price of gold per gram [16]. The high economic value of *O. sinensis* has driven the global cordyceps consumer market to exceed tens of billions of US dollars [17]. In addition to boosting the international consumer market, *O. sinensis* plays a key role in poverty alleviation in rural production areas [18]. In 2013, sales in the Tibet Autonomous Region alone reached US $1.2 billion [15], with *O. sinensis* income accounting for 50–80% of the total income for local farmers and herders [13]. Surveys conducted in rural Nepal from 2015 to 2018 indicated that the harvesting and sale of the fungus constituted a primary source of cash income for local communities [18]. However, while *O. sinensis* has stimulated rural economies, it also carries risks; in 2007, eight Tibetan villagers were killed in conflicts over its collection [19]. The enormous market demand, coupled with overharvesting and habitat destruction, has raised serious sustainability concerns. *O. sinensis* is now listed on the IUCN Red List of Threatened Species [20], and it is subject to strict regulations in several producing countries [14,21,22]. Thus, developing efficient and sustainable artificial cultivation technologies has become a crucial pathway to alleviate the supply-demand conflict and protect wild resources [3,22].

After half a century of research, artificial cultivation in the *Cordyceps* industry has achieved breakthroughs [22,23]. Firstly, *H. sinensis*, recognized as the anamorph (asexual stage) of *O. sinensis*, has been successfully isolated from *O. sinensis* and cultivated on a large scale artificially [15,24]. Additionally, other fungi isolated from *O. sinensis*, such as *Cephalosporium sinensis* Chen, *Clonostachys rosea* Bainier [*Clonostachys rosea* (Link) Schroers, Samuels, Seifert & W. Gams], *Mortierella hepialid* C.T. Chen & B. Liu, and *Paecilomyces hepialid* Q.T.Chen & R.Q.Dai, have been developed into numerous pharmaceutical products [13,25]. Secondly, studies have shown that over 50 species of *Hepialus* and *Thitarodes* moths can serve as primary potential hosts for *H. sinensis* [10]. Completing the life cycle of these host insects requires over five years in nature, but artificial rearing has significantly shortened the larval generation time by nearly half [26]. Furthermore, artificial cultivation has been largely achieved for other cordyceps species, such as *Cordyceps militaris* (L.) Fr., *Ophiocordyceps hawkesii* (G. Cunn) G.H. Sung, J.M. Sung, Hywel-Jones & Spatafora, *Ophiocordyceps xuefengensis* T.C. Wen, K.D. Hyde & X.G. Zhang, and *Ophiocordyceps gracilis* (Grev.) G.H. Sung, J.M. Sung, Hywel-Jones & Spatafora [15,20,27]. Based on these species, a multitude of drugs for treating human diseases have been derived [13,25].

Currently, artificial cultivation of *O. sinensis* primarily relies on two strategies: in vitro cultivation and in vivo inoculation [15,24]. Although significant progress has been made with both approaches, each still faces key technical bottlenecks. In vitro cultivation, aimed at large-scale production of mycelia, primarily involves techniques like solid-state fermentation (SSF) and liquid fermentation (submerged and surface fermentation) [20]. While these methods can efficiently obtain bioactive compounds from *O. sinensis*, they still involve complex operational processes, are susceptible to cross-contamination, and currently, large-scale cultivation is only suitable for a limited number of easily cultivated *Cordyceps* species [20]. On the other hand, in vivo inoculation technology also has apparent limitations. Although artificial rearing has been achieved for over 14% of potential host larvae, their long-term breeding still faces degenerative issues such as skewed sex ratios and decreased fecundity [28]. Simultaneously, the infection efficiency of *H. sinensis* against *Hepialus* and *Thitarodes* larvae is low, induction of stromata formation is difficult, and the mechanisms underlying fungus-host interactions remain unclear [24]. Therefore, a systematic and in-depth analysis of the key stages in existing artificial cultivation methods for *Cordyceps* is of great significance for further breakthroughs in breeding technologies.

To address the acute contradiction between the depletion of wild *O. sinensis* resources and growing market demand, this review systematically summarizes the two main artificial cultivation strategies—in vitro cultivation and in vivo inoculation—analyzes their key technical bottlenecks and future prospects, and provides a theoretical basis for achieving chemical consistency between artificially cultivated and wild *O. sinensis*. We first outline the resource distribution and market status of *O. sinensis*, then compare the core chemical composition differences between wild and cultivated materials, and finally focus on discussing the technical advantages, challenges, and potential breakthroughs for the two cultivation strategies.

## 2. Depletion of Wild *O. sinensis* Resources and Soaring Market Demand

### 2.1. Distribution of Wild O. sinensis and Multiple Drivers of Its Decline

*O. sinensis,* the most representative high-value species within the Cordyceps genus, has wild resources primarily concentrated in four countries: China, Bhutan, India, and Nepal [20,29]. It is noteworthy that some scholars posit that only the *O. sinensis* produced in the alpine meadow regions of the Qinghai–Tibet Plateau qualifies as authentic wild Chinese cordyceps [23,30].

**Figure 1:**
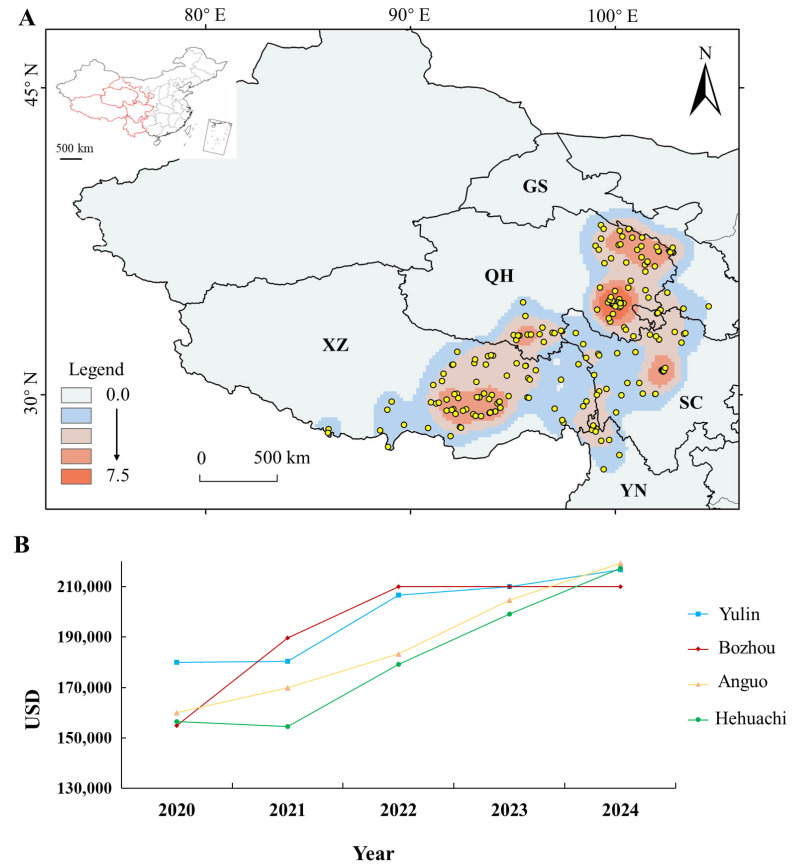
Distribution and Price Trends of *O. sinensis*. (**A**) Kernel density of *O. sinensis* resource distribution. Dots represent recorded occurrence points, with color intensity indicating local population density. XZ, Tibet Autonomous Region; QH, Qinghai Province; YN, Yunnan Province; SC, Sichuan Province; GS, Gansu Province. (**B**) Price variation of *O. sinensis* in four major Chinese herbal medicine markets from 2020 to 2024. Data points reflect the average price across multiple producing areas for each market. Source data were obtained from the Zhongyacao Tiandi Network (https://www.zyctd.com/ (accessed on 1 January 2025). Yulin, Yulin Market (Yulin City, Guangxi Province, China); Bozhou, Bozhou Market (Bozhou City, Anhui Province, China); Anguo, Anguo Market (Anguo City, Hebei Province, China); Hehuachi, Hehuachi Market (Chengdu City, Sichuan Province, China).

The global annual production of wild *O. sinensis* is approximately 83.2–182.5 tons, with China accounting for over 95% of the total output [24]. However, compared to the production level of around 1 million kilograms in the 1950s, the yield in China has plummeted by more than 50% in recent years [27]. Within China, wild *O. sinensis* resources are mainly distributed across provinces and autonomous regions including Tibet, Qinghai, Gansu, Sichuan, and Yunnan (Figure 1A), covering about 10% of the country’s land area [24,31]. In terms of physical geography, its distribution within China generally extends north to the Qilian Mountains, south to the high mountains of northwestern Yunnan, east to the western Sichuan plateau, and west to most areas of the Himalayas [32]. Among these regions, Tibet and Qinghai not only have the highest yields [5,31] but also produce wild *O. sinensis* considered to be of the highest quality nationally [23]. As a type of environmental product, *O. sinensis* holds significant importance for improving livelihoods and poverty reduction in low- and middle-income regions [33].

Although *O. sinensis* resources have played a pivotal role in poverty alleviation in mountainous areas, the income generated from them is susceptible to fluctuations in yield [18]. The decline in yield is primarily attributed to climate change and overharvesting [14], although the dominant factor exhibits regional variation: overharvesting is the main driver in Qinghai, whereas climate change is a more significant factor in Tibet and Sichuan [20,29].

Temperature and precipitation are key climatic factors influencing the distribution of *O. sinensis* [17]. Larger-sized *O. sinensis* are mostly distributed in areas above 4600 m in altitude, with approximately 3000 m representing the lower distribution limit [24]. Increased snowfall in early spring can enhance yield within the same year [5]; however, the rising snow line caused by global warming may lead to spatial shifts in the productivity of various producing regions [17]. Most studies indicate that climate warming will lead to a contraction of the suitable habitat for *O. sinensis* [14], but this change may exhibit regional heterogeneity [18]. For instance, climate model projections suggest that the yield of *O. sinensis* in Nepal may increase with climate change, while it may decrease in Tibetan producing regions [34]. The daily average collection quantity in upstream areas of Gorkha District showed an increasing trend from 2015 to 2018 [18], indicating that localized suitable habitats may be shifting to higher altitudes [17]. Therefore, despite the lack of direct evidence, a cautiously optimistic attitude regarding the global supply of *O. sinensis* resources can still be maintained [35].

Besides overharvesting and climate change, the protracted life cycle of its host insect—which typically requires more than 1000 days to complete—also contributes to the decline in *O*. *sinensis* resources [24,36]. The distribution of *O. sinensis* is closely linked to that of its host insect, exhibiting prolonged zonation, regional distribution, and vertical stratification, with food availability being a primary limiting factor for the insect [5]. Consequently, environmental changes in recent years may have altered the distribution of food sources, further reducing the survival of *O. sinensis* after its extended development. Moreover, overgrazing in pastoral areas can degrade the herbaceous layer of alpine meadows, leading to decreased abundance or diminished quality of the fungus [27].

To protect *O. sinensis* resources, several countries have strengthened conservation through legislation, further limiting its production. Bhutan has listed *O. sinensis* in Schedule I of its Forest and Conservation Act since 1995 [37]; conversely, the Indian state of Uttarakhand has classified it as a non-timber forest product under the Indian Forest Act of 1927 and formulated specific guidelines for its collection and trade [38].

The declining supply of wild *O. sinensis* and the growing market for artificially cultivated cordyceps have prompted comparisons between wild and cultivated *O. sinensis* among many consumers and scholars [23].

### 2.2. Market Value and Demand

*O. sinensis* is harvested in multiple provinces surrounding the Qinghai–Tibet Plateau. The highest quality specimens, which consequently fetch the highest market prices, are sourced from Naqu, Tibet, and Yushu, Qinghai [39,40,41]. Since the early 1970s, the price of *O. sinensis* has increased several hundred-fold from approximately 20 RMB per kilogram [16], reaching up to 140,000 USD per kilogram for premium grades by 2017 [20,29]. In recent years, its price in China’s four major medicinal markets has continued to climb (Figure 1B), indicating sustained, robust market demand for high-quality wild *O. sinensis*.

The exceptionally high and rising economic value of *O. sinensis* has fostered a unique rural fungal economy in the high-altitude regions of the Qinghai–Tibet Plateau [24]. Sales in the Tibet Autonomous Region alone reached 1.2 billion USD in 2013 [15]. Similarly, in the Upper Gorkha region of Nepal, *O. sinensis* harvesting generated 614,000 USD in income for several hundred impoverished households in 2018 [18]. The substantial market value of *O. sinensis* is likely driven by multiple factors.

Firstly, the harsh growth environment contributes to its high value. Wild *O. sinensis* is distributed in alpine meadows above 3000 m on the Qinghai–Tibet Plateau [23], with larger specimens typically found at higher altitudes [24]. The Plateau is characterized by scarce flora and fauna, intense ultraviolet radiation, low oxygen levels, and limited arable land [32,42]. These challenging living conditions, coupled with the lack of farming opportunities, result in a low average population density of approximately 5 people per km^2^ [42]. Beyond the extreme environment, the purported health benefits and regulatory status of *O. sinensis* also enhance its economic appeal. Unlike many traditional Chinese medicines classified strictly as pharmaceuticals, *O. sinensis* is often categorized as a food or dietary supplement component [11]. This classification allows it to be marketed in many countries, including those in Europe and North America, without undergoing stringent safety and efficacy approvals required for drugs [43]. Its value is further amplified by its diverse perceived pharmacological effects. Firstly, due to the absence of banned substances and significant side effects, it is used to alleviate fatigue and enhance athletic performance [44]. The polysaccharides present in *O. sinensis* exhibit antioxidant properties and have been reported to significantly benefit female wrestlers [45] and long-distance runners [8]. Secondly, bioactive compounds such as cordycepin and ISP-1 (a substance structurally similar to sphingosine) have drawn attention for their potential to reduce organ transplant rejection [6]. These primary active components are being investigated for managing transplant rejection, systemic lupus erythematosus, and other immune-related conditions [46]. For instance, application of *O. sinensis* extract in clinical penetrating keratoplasty demonstrated significant immunosuppressive effects, reducing rejection rates while improving graft transparency [10]. Similarly, Bailing capsules, made from *O. sinensis* extract, effectively prevent allograft rejection [47]. Furthermore, bioactive components like cordycepin, ergosterol, and polysaccharides show notable anti-cancer effects [48]. These substances are thought to inhibit cancer cell proliferation through mechanisms including apoptosis induction, cell cycle arrest, and modulation of key signaling pathways [48]. An extract from *C. sinensis* fruiting bodies (WECS) significantly stimulated A3-R and activated GSK-3β, exerting cytotoxic effects on B16-BL6 and LLC cells [49]. Administration of WECS at 100 and 200 mg/kg for two weeks significantly reduced tumor volume by 28% and 43%, respectively [50]. Finally, *O. sinensis* is traditionally used to treat asthma, cough, and impotence [48]. The main bioactive components and their efficacies of Cordyceps sinensis are summarized in Table 1. The combination of its diverse applications, limited geographical distribution resulting in low natural abundance, and low probability of natural inoculation likely collectively drive its high price.

The high value and economic potential of *O. sinensis* have spurred the development of its artificial cultivation. However, this has also raised concerns regarding potential differences in active components between wild and cultivated specimens. Wild *O. sinensis* primarily contains compounds such as cordycepic acid (mannitol), adenosine, ergosterol, and various bioactive polysaccharides [24]. Several studies indicate no significant differences in certain components: Zan et al. [52] reported that adenosine levels in both wild and cultivated *O. sinensis* meet the standards of the Chinese Pharmacopoeia (2015 edition). Guo et al. [60] found largely consistent sterol profiles and ergosterol content. Li et al. [61] developed a detection method for cordycepic acid showing no significant difference in its content. Despite these similarities in some active constituents, notable differences exist in specific marker compounds. Wild *O. sinensis* tends to contain higher levels of adenosine, mannitol, and trehalose [62,63], whereas cultivated *O. sinensis* often contains more amino acids and myo-inositol [23,64,65]. Additionally, significant differences in total arsenic content can serve as a distinguishing marker [66].

Nevertheless, the surging demand for *O. sinensis* fruiting bodies and their bioactive compounds makes sustainable cultivation a primary strategy to bridge the supply-demand gap, despite existing discrepancies in certain active components between cultivated and wild specimens. Consequently, advancing the understanding of its cultivation protocols is an essential step toward minimizing these qualitative differences.

## 3. Two Approaches to the Artificial Cultivation of *O. sinensis*

**Figure 2:**
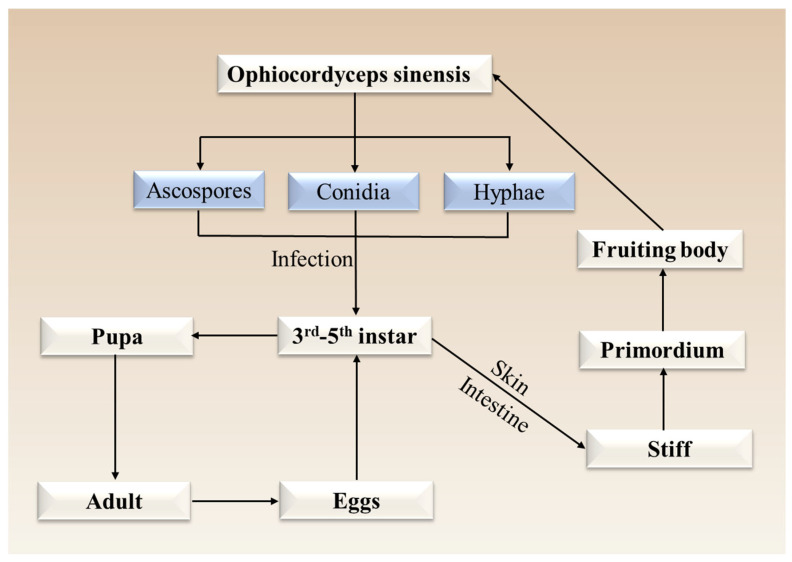
The complete life cycle of *O. sinensis*.

*O. sinensis* retains the structural form of an insect host while embodying the vitality of a fungus [3]. It specifically refers to the dried composite body, composed of the fungal stroma and sclerotium, that forms after the fungus *Ophiocordyceps sinensis* infects larvae of insects belonging to the genera *Hepialus* and *Thitarodes* [23]. The development of this complex structure requires the fungus to complete both teleomorph and anamorph stages, and is further dependent on the protracted and complex life cycle of its host insect (Figure 2).

During summer, spores of *O. sinensis* are randomly released into the topsoil, where they mature and are carried by rainwater into deeper soil layers [29]. In late autumn, ascospores, conidia, or hyphae infect larvae of Lepidoptera, such as genera *Hepialus* and *Thitarodes*, via the cuticle or intestinal tract [24,67]. Larvae at the 4th–5th or 3rd–4th instar molting stages exhibit the highest susceptibility to infection, whereas larvae at other stages are less vulnerable [24]. After infection, the fungal cells proliferate by yeast-like budding in the hemocoel, eventually filling the host’s body cavity [65,67,68]. The fungus then manipulates the host to move upward to a soil depth of 2–5 cm, where the larva dies with its head oriented upward [69]. Fungal proliferation leads to energy depletion as a primary cause of host death, and hyphae spread throughout the circulatory system, consuming all internal larval tissues except the exoskeleton [29]. Additionally, fungal infection may alter host metabolism, which could be a key factor inducing this behavioral manipulation [30]. Before soil freezing, a small stroma bud emerges from the head of the sclerotium (the mummified host larva). In the following spring, the stalked fruiting body elongates and breaks through the soil surface, developing a fertile head bearing mature perithecia containing thread-like ascospores [24,69]. During host colonization, *O. sinensis* produces secondary metabolites at each developmental stage, potentially as countermeasures against host immune defenses [20].

Within the complex life history of *O. sinensis*, the availability of host insects and the development of the fruiting body are key factors influencing its successful completion. Consequently, artificial cultivation efforts have diverged into two distinct directions, each addressing these critical bottlenecks. However, it is noteworthy that current advanced techniques in its cultivation remain largely confidential and are not publicly disclosed.

### 3.1. Mycelial Fermentation

#### 3.1.1. Fungal Species

According to the current taxonomic system, *O. sinensis* belongs to the phylum Ascomycota, class Sordariomycetes, order Hypocreales, family Ophiocordycipitaceae, and genus *Ophiocordyceps* [70]. The genus *Cordyceps* comprises over 750 identified species globally, with approximately 200 species reported in China [71]. Among these, *O. sinensis* is one of the most prominent representatives [8]. This fungus is psychrophilic, exhibits extremely slow growth, and thrives within a temperature range of 4–21 °C, with an optimum between 15 and 18 °C [72]. Its developmental process can be divided into three distinct phases: the blastospore stage, the pseudohyphal stage, and the hyphal stage [3].

The taxonomic study of Chinese *O. sinensis* began with its initial naming by the Italian scholar Saccardo in 1878, followed by subsequent classifications of *Cordyceps* fungi in China by Teng [73]. To date, 125 valid species, three varieties, and two forms of Cordyceps have been documented in China [74], and this number continues to grow, although research remains largely focused on *O. sinensis* and closely related species [73].

Since the 1970s, Chinese researchers have isolated more than twenty fungal strains from natural *O. sinensis* fruiting bodies that are associated with its teleomorph [75]. These include *H. sinensis*, *P. hepialid*, *M. hepialid*, *C. sinensis*, and *C. rosea*, among others [24]. Among these, *H. sinensis* was formally described in 1989 [76] and has been confirmed as the sole true anamorph of *O. sinensis* based on Koch’s postulates [24]. Consequently, *O. sinensis* has two recognized names: *O. sinensis* and *H. sinensis*. However, in line with the “One Fungus, One Name” principle, the species will eventually be referred to solely as *O. sinensis* [77].

As an ascomycete, the sexual reproduction of *O. sinensis* is governed by a single mating-type locus (MAT1) [78], which, in isolates with the MAT1-1 idiomorph, is characterized by the presence of three genes—*MAT1-1-1*, *MAT1-1-2*, and *MAT1-1-3*—indicating a homothallic breeding system [79]. This multi-gene arrangement contrasts with other species in the broader *Cordyceps* sensu lato group, such as *Tolypocladium inflatum* W. Gams [80] and *C. militaris* [81], which possess only a single mating-type gene at the locus.

*O. sinensis* initially grows parasitically within a living larva and transitions to a saprophytic phase after the host’s death, indicating that it is not an obligate biotroph but rather a facultative saprophyte [24]. Conidia serve as the primary infectious propagules. Therefore, the isolation and identification of a substantial number of conidia constitute a critical prerequisite for achieving successful artificial cultivation [24,82].

#### 3.1.2. Industrial Fermentation Production

Mycelia of *O. sinensis*, obtained through low-temperature fermentation of isolates from natural *O. sinensis*, serve as a substitute for wild-harvested material [23]. Common isolation techniques include tissue separation and ascospore isolation methods [5,83].

Industrial fermentation offers advantages such as shorter production cycles, standardized and controllable processes, and adjustable yields of key secondary metabolites [23]. The predominant cultivation strategies are solid-state fermentation (SSF) and submerged fermentation. SSF involves inoculating the fungus onto solid organic substrates (e.g., corn, soybeans), with cultivation progressing through three stages: mycelial development, fruiting body formation, and accumulation of active compounds like cordycepin. While this method more closely mimics the natural environment, it is often labor-intensive and difficult to scale. Submerged fermentation, typically conducted in bioreactors, is suitable for large-scale mycelial production. This approach offers shorter cycles and consistent output quality but carries a risk of strain degeneration [20,29].

The formation of conidia, blastospores, and mycelia can be observed in both fermentation systems [5]. Temperature, dissolved oxygen, pH, and nutrient composition are critical parameters influencing mycelial growth. Considerable research has focused on optimizing culture media to enhance the yield of target metabolites. For instance, Zhang et al. [84] achieved a conidial density of 1.0 × 10^4^ conidia/g by solid-state fermentation at 18 °C for 30 days using peat soil supplemented with a nutrient solution. Ge et al. [85] utilized a solid medium comprising 50% rice, 20% corn flour, 20% wheat bran, and 10% silkworm pupa powder to obtain mycelia rich in ergosterol.

The optimal growth temperature for *O. sinensis* is 18–20 °C, with growth ceasing above 25 °C. The preferred pH range is 5–6. Glucose serves as the optimal carbon source, peptone as the nitrogen source, and yeast extract can further promote growth. Trace inorganic salts such as KH_2_PO_4_ and MgSO_4_ also enhance mycelial development [5]. However, most current parameter optimizations are based on *H. sinensis*, overlooking the potential role of other symbiotic fungi within the native *O. sinensis* system. This oversight may partly explain the chemical compositional differences between artificially cultured mycelia and wild-type materials.

Currently, more than ten health food products primarily derived from fermented mycelia of *O. sinensis* have been approved for the market in China [13,83]. The development of the *O. sinensis* industry has also stimulated advances in related sectors involving other *Cordyceps* fungi. Although other species within the genus *Cordyceps* cannot fully substitute for the value of *O. sinensis*, they provide valuable references for its future industrial development. In China, only a few wild *Cordyceps* species are capable of forming fruiting bodies [28]. Since the successful cultivation of *C. militaris* fruiting bodies in 1867, other species including *Cordyceps norvegica* Johan-Olsen, *Cordyceps pruinosa* Petch, and *Cordyceps tenuipes* (Peck) Kepler, B. Shrestha & Spatafora have also been artificially cultivated [86]. For some *Cordyceps* species that are difficult to fully develop into fruiting bodies, their fermented mycelia still exhibit medicinal potential [87]. As of 2023, fruiting bodies of 40 species within the genus *Cordyceps* have been successfully cultivated; detailed taxonomic information can be found in Li et al. [28].

Although multiple *Cordyceps* species have been artificially cultivated, only *C. militaris*, *C. chanhua* and *C. guangdongensis* have achieved commercial success alongside *O. sinensis* [15,88,89] (Table 2) These species share certain chemical similarities with *O. sinensis* but differ in their geographical distributions *C. militaris* exhibits faster growth and is more amenable to artificial fruiting body formation compared to *O. sinensis*. China is the first country to achieve large-scale artificial cultivation of *C. militaris* fruiting bodies using insect pupae, such as those of the silkworm (Bombyx mori) or Chinese oak silkworm (*Antheraea pernyi*) [13]. *C. militaris* has now been industrialized and was approved as a novel food ingredient by the former Ministry of Health of China in 2009 [90]. *C. chanhua*, recognized for its anti-fatigue, sleep-improving, and renal function-enhancing properties, can be artificially cultivated for synnemata production and has achieved large-scale industrial manufacturing [13]. *C. guangdongensis* forms fruiting bodies through infection of the fungus *Elaphomyces* by *C. guangdongensis* and is currently found only in Guangdong Province. It possesses notable health benefits and high edible safety, leading to its approval as a novel resource food in 2013 [91] and the realization of industrial-scale cultivation [13]. Although these *Cordyceps* species exhibit certain chemical and pharmacological similarities to *O. sinensis* [70], they display distinct biological characteristics and metabolic profiles, and therefore should not be considered fully equivalent.

Beyond issues of species authenticity, products derived from in vitro cultured *O. sinensis* may contain culture medium residues, such as protein-based allergens. Allergic reactions to *Cordyceps* products have been reported, linked to the presence of undissolved silkworm pupae [92]. Therefore, controlling and conducting safety evaluations of cultivation residues are critical foci for future research.

**Table 2:** Commercially cultivated *Cordyceps* species.

Species	Wild Distribution	Chemical Constituents	References
*Ophiocordyceps sinensis*	Tibet, Qinghai, Sichuan, Yunnan, and Gansu provinces	Adenosine, cordycepin, mannitol, polysaccharides, ergosterol, glutamic acid, arginine, tryptophan, tyrosine, trace elements, vitamins	[8,9]
*Cordyceps militaris*	Yunnan, Guizhou, Sichuan, Chongqing	Adenosine, cordycepin, N6-(2-hydroxyethyl)-adenosine, pentostatin, adenine, 2′-deoxyuridine, mannitol, polysaccharides, albumin, glutelin, globulin, γ-aminobutyric acid (GABA), ergothioneine, lovastatin, sterols, cerebroside B	[4,93,94,95,96,97,98]
*Cordyceps chanhua*	Zhejiang, Jiangsu, Anhui, Hubei, Hunan, Guangdong, Sichuan, Yunnan, Fujian, and Taiwan	Polysaccharides, nucleosides, mannitol, ergosterol, myriocin, amino acids	[99]
*Tolypocladium guangdongense*	Guangdong	Adenosine, cordycepin, mannitol, glutamic acid, arginine	[100,101]

### 3.2. Bio-Cultivation of O. sinensis

#### 3.2.1. Host Insect Species of *O. sinensis*

The unique growth cycle and protracted development of *O. sinensis* complicate the study of its host insects [14]. Historically, taxonomic classifications of these hosts have varied, reflecting the technological and methodological constraints of different research periods. The initial discovery and naming of a hepialid moth associated with *O. sinensis* date back to 1886, when a British scholar identified a specimen from Baoxing County, Sichuan Province, as *Hepialus davidi* Candèze [5]. However, the accuracy of this designation has been questioned due to inconsistencies between the original name and the corresponding specimen [102]. Subsequent fieldwork by Chinese researchers across various regions established the genus *Hepialus* as the primary host of *O. sinensis* [102,103].

In a parallel taxonomic effort, the French entomologist Oberthür described an adult moth from a medicinal material collection as *Hepialus armoricanus* Oberthür [104]. Later, Viette [14] designated *H. armoricanus* as the type species for the new genus *Thitarodes*, based on morphological characteristics of the male genitalia [104]. Nielsen et al. [105] subsequently proposed reassigning nearly all *Hepialus* species reported after 1984 to the genus *Thitarodes*. Further refining this classification, Zou et al. [106] revised the taxonomy of Chinese hepialid moths based on male genital structures, establishing the genera *Parahepialus* Chu & Wang and *Ahamus* Viette, and reclassifying 60 Chinese species into *Parahepialus* (1 species), *Ahamus* (18 species), *Hepialus* (1 species), and *Thitarodes* (40 species).

Reflecting these taxonomic revisions, several studies have sought to identify the potential host insects of *O. sinensis*. Wang and Yao [107] analyzed previously reported host species and concluded that, of 91 insect species across 13 genera, only 57 were likely true hosts. These were distributed across the genera *Bipectilus* Chu & Wang (1 species), *Endoclita* Felder & Rogenhofer (1), *Gazoryctra* Hübner (1), *Hepialus* (12), *Magnificus* (2), *Pharmacis* (3), and *Thitarodes* (37). Based on data from multiple *O. sinensis* production areas, Qiu et al. [31] identified *Thitarodes* (37 species), *Ahamus* (16 species), and *Hepialus* (7 species) as the principal host genera, noting that the dominant species varies by geographic region. Despite the diversity of potential hosts, their artificial cultivation faces significant challenges. First, only a limited number of these species have been successfully reared in the laboratory. To date, successful cultivation has been reported for just eight species: *Thitarodes oblifurcus* Chu & Wang, *Thitarodes baimaensis* Liang, *Thitarodes menyuanicus* Chu & Wang, *Thitarodes lagii* B. Péter, *Hhitarodes gonggaensis* Fu & Huang, *Thitarodes jianchuanensis* Yang, *Thitarodes xiaojinensis* Liang, and *Thitarodes luquensis* Yang & Yang [26]. Second, wild host insect populations may be experiencing degradation and can be difficult to infect successfully. Adult hepialid moths have short lifespans (3–8 days), do not feed, and are poor dispersers, limiting gene flow between populations and species. Geographical and climatic barriers further restrict genetic exchange among populations [5].

Therefore, a thorough understanding of the biology and ecology of known host insects is essential for identifying new, easily cultivable host resources for the future artificial cultivation of *O. sinensis*.

#### 3.2.2. Rearing of Host Insects

Hepialid moths are holometabolous insects, undergoing a life cycle that includes egg, larval, pupal, and adult stages [24]. Their development is constrained by numerous environmental factors, typically requiring 3 to 6 years to complete, with the larval stage being the longest [5]. For instance, the life cycle of *Thitarodes puilarva* spans 1095 to 1460 days, of which the larval stage accounts for 990 to 1350 days and involves 7 to 9 instars [32]. Beyond their protracted development, survival rates under natural conditions are influenced by multiple factors and are generally less than 10% [36].

Since the beginning of this century, significant breakthroughs in artificial rearing at low altitudes have nearly halved the life cycle duration of these moths [5]. Under controlled conditions, key focuses of cultivation, besides simulating the plateau climate, include optimizing larval diet and preventing cannibalism in larvae beyond the third instar [24,26,108].

Larvae of *O. sinensis* host insects live in underground tunnels in the wild and are omnivorous, primarily feeding on tender plant roots. Their diet includes the young fibrous roots and bulbs of 39 plant species from 31 genera across 19 families, such as Cyperaceae, Polygonaceae, Gramineae, and Gentianaceae [5,109]. In a Y-tube olfactometer experiment with second-instar larvae of *H. armoricanus*, Wang et al. [47] observed varying olfactory preferences towards different plants, suggesting that olfaction guides their foraging behavior. Stable carbon isotope analysis of hepialid larvae from the Sejila Mountain region on the Qinghai–Tibet Plateau further indicates the existence of two dietary groups: one primarily consuming soil humus and the other feeding mainly on tender plant roots [5,26].

Artificial diets for these larvae are broadly categorized into practical and semi-purified feeds [109]. Strategic diet formulation can promote larval growth. For example, Shen et al. [110] reported that feeding larvae carrots in September and October increased their average body weight by 27.75% compared to feeding them *Polygonum viviparum*. Huang et al. [111] found that using Chinese cabbage leaves and carrots helped regulate humidity control. Supplementing the diet of *H. gonggaensis* with the *Carnobacterium* sp. strain Hg4-03 significantly enhanced larval digestive enzyme activity and promoted growth and development [112]. Overall, the effects of different artificial diet combinations vary, and the optimal formulation should be selected based on the specific biological characteristics of the target host insect.

Besides diet composition, artificial rearing methods for potential *O. sinensis* hosts primarily fall into three categories. The first is Petri dish rearing. This method is straightforward, suitable for small-scale operations and various experiments, but it is relatively costly [113]. The second involves indoor rearing with the release of a moderate number of larvae onto turf-covered substrates to simulate a natural ecological environment [114]. While this approach allows for increased rearing density, it can lead to cannibalism, hindering high-density cultivation [115]. The third method is simulated natural ecological stocking. This is conducted in enclosed plots (to prevent insect escape) planted with host-preferred plants, into which a controlled number of insects are released [115]. This method supports large-scale, high-density rearing but involves relatively high costs [113].

A significant challenge in current artificial cultivation is the degradation of successive generations of *Hepialus* larvae, which constrains industry development. In *T. xiaojinensis*, issues such as atrophy and malformation of the male seminal vesicle can lead to a decline in offspring metrics, including body weight, fecundity, and lifespan [116]. Nevertheless, the population can remain sustainable for at least two generations under managed conditions [117]. Beyond larval degradation, the low success rate of infection by *O. sinensis* is another major bottleneck. Direct observation of the infection process in the high-altitude habitat is challenging, but two primary hypotheses exist. First, during the larval molting stage, conidia may infect larvae through the vulnerable new cuticle or via spiracles [118]. Alternatively, larvae might ingest conidia attached to plant roots during feeding, leading to infection through the digestive system [119].

In *T. xiaojinensis* larvae infected by *O. sinensis*, the fungal load is highest in the fat body, followed by the body wall, haemolymph, and intestinal wall [120]. Numerous attempts have been made to infect host larvae with entomopathogenic fungi (Table 3). Wang et al. [121] inoculated 4th-6th instar larvae using suspensions of ascospores, conidia, or mycelia applied via needle puncture, smearing, feeding, immersion, or spraying. Their results indicated that smearing and spraying methods each achieved a 15% infection rate. Tu et al. [122,123] tested various inoculation methods on different larval instars and found that mixing the fungal inoculum into the diet yielded the highest infection rates (80.77% and 80.10%, respectively). The most susceptible instars were the 3rd and 4th, and successful infection required over 50 days post-inoculation.

Achieving industrial-scale production requires not only the selection of highly virulent, genetically stable *O. sinensis* strains and the mass rearing of hepialid insects but also meeting the critical benchmarks of high larval infection rate, high mummification rate, and high primordium formation rate [31,124]. The formation of the *O. sinensis* primordium is likely linked to specific ecological factors of the unique alpine ecosystem on the Qinghai–Tibet Plateau. Once primordium induction is successfully achieved, subsequent fruiting body development generally proceeds with relative ease [24].

**Table 3:** Methods for inoculating host larvae with *O. sinensis*.

Inoculation Method	Procedure	References
Cuticular Infection	Topical application of suspensions containing ascospores, conidia, or mycelia.	[125,126]
	Infection of larvae with fungal mycelial mats.	[46]
	Laser-induced micro-abrasions followed by spore application.	[47]
Oral Infection	Feeding larvae with symbiotic complexes of *H. sinensis* (the anamorph of *O. sinensis*) and plant tissues.	[127]
Internal Injection	Direct micro-injection of mycelial and conidial suspensions using a needle	[128]
	Injection of blastospore mixtures.	[129]

Following successful infection by the entomopathogenic fungus, a suite of molecular and biochemical changes are initiated to facilitate the manipulation of the host. Mannitol has been identified as playing a crucial role in fungal development and the manipulation of host behavior [3]. The apolipophorin III (apoLp-III) protein appears to be involved in the immune response of hepialid moths against fungal infection [130]. A comprehensive study on *Thitarodes jiachaensis* larvae demonstrated that the infection by *O. sinensis* elicits responses from various host factors, including cuticle proteins, peritrophic matrix proteins, antimicrobial peptides (AMPs), pattern recognition receptors (PRRs), and hydrolases [131]. Transcriptomic analysis of *T. puilarva* larvae reared at different altitudes revealed significant differential expression of genes related to carbohydrate and lipid metabolism, as well as respiration [68].

Notably, the complete life cycle of *O. sinensis*, including the formation of fruiting bodies and sexual reproduction, has been successfully achieved using the host *T. xiaojinensis* under controlled conditions [132]. This breakthrough provides a critical reference for selecting new potential host insects and for future research into the intricate interactions between hepialid moths and entomopathogenic fungi.

## 4. Conclusions and Outlook

*O. sinensis*, a prized medicinal resource renowned as “soft gold”, is caught in a critical conflict between the depletion of its wild populations and a surging global demand. This review has systematically delineated the multiple dimensions of this challenge. The wild resource, dependent on the unique alpine ecosystem of the Qinghai–Tibet Plateau, has a narrow and fragile distribution. Under the combined pressures of overharvesting and climate change, its population size and suitable habitat area are continuously diminishing, warranting its classification as an endangered species. Furthermore, its exceptionally high market value, while supporting local economies, intensifies this unsustainable trajectory.

In this critical context, artificial cultivation emerges as a vital pathway for both resource conservation and sustainable utilization. We have critically compared the two primary technological strategies: in vitro mycelial cultivation and in vivo inoculation. Our analysis indicates that in vitro fermentation, with its short cycle and ease of standardization, has become the primary source of alternative bioactive compounds. However, the chemical profile of its products differs from that of wild fruiting bodies, and a significant quality gap remains. While in vivo inoculation can yield fruiting bodies more comparable to their wild counterparts, it is hampered by a series of complex biological bottlenecks. These include the challenges of mass-rearing host insects, the rapid degeneration of successive insect generations, and low fungal infection rates, which collectively result in low productivity and high costs.

Consequently, the future sustainable development of the *O. sinensis* industry must rely on a dual strategy integrating stringent conservation and targeted innovation:

Strengthening wild resource protection and management. Immediate implementation of science-based harvesting quotas and active ecological restoration programs in production areas is imperative. Establishing a dynamic resource monitoring network represents a fundamental strategy to alleviate the immediate crisis and buy crucial time for artificial cultivation technologies to mature.

Focusing on breakthroughs in core cultivation technologies. For in vitro cultivation, research should focus on metabolic engineering and the optimization of fermentation processes to directionally enhance the yield of key bioactive compounds, striving to bridge the efficacy gap with wild-collected specimens.For in vivo inoculation, the core challenges lie in overcoming the technological barriers to sustainable host insect rearing and gaining a deeper molecular-level understanding of the fungus–insect interaction. This fundamental knowledge is key to improving infection rates and the efficiency of fruiting body induction.

Establishing a comprehensive quality standard system. Developing a multi-dimensional quality assessment framework that integrates chemical fingerprinting with biological activity assays is essential. This will help regulate the market, clearly differentiate between wild and cultivated products, and guide the industry toward higher quality and transparency.

Ultimately, the future of *O. sinensis* hinges on leveraging technological breakthroughs in artificial cultivation to meet market demand, thereby creating the necessary space for the recovery of wild populations. Only by synergizing cutting-edge biotechnology with robust field conservation can this invaluable natural heritage be secured against the threat of extinction, ensuring the sustainable preservation of both its ecological and economic value.

## Figures and Tables

**Table 1:** Main bioactive components of *C. sinensis*.

Component	Category	Biological Functions	References
Polysaccharides	Polysaccharides	Immune modulation; tumor therapy; anti aging; endocrine regulation; improvement of athletic performance	[8,45]
Soluble polysaccharides			[51]
Adenosine	Nucleosides	Vasodilation, lowering of blood pressure; reduction in heart rate; other important pharmacological effects	[52]
Inosine			[53]
Hypoxanthine			[53]
Cholesterol	Sterols	Immune modulation; anti tumor; anti aging; enhancement of lung function; inhibition of cell proliferation	[54]
Ergosterol			[55]
Sitosterol			[55,56]
Cordycepin	Adenosine derivatives	Reduction in organ rejection; antibacterial; anti inflammatory; antiviral; anti tumor and immunomodulatory activities	[6,46]
Cordycepic acid (Mannitol)	Alcohols	Inhibition of tumors and enhancement of immunity	[57]
Polyphenols	Polyphenols	Strong antioxidant and potential anti cytotoxic activities	[58,59]

## Data Availability

No new data were created or analyzed in this study. Data sharing is not applicable to this article.
